# The Anatomical and Functional Outcomes of 27-Gauge Pars Plana Vitrectomy in Diabetic Tractional Retinal Detachments in the South Asian Population

**DOI:** 10.7759/cureus.38099

**Published:** 2023-04-25

**Authors:** Muhammad A Awan, Fiza Shaheen, Fatima Mohsin

**Affiliations:** 1 Ophthalmology, Shifa International Hospital, Islamabad, PAK; 2 Ophthalmology, Shifa Tameer-E-Millat University Shifa College of Medicine, Islamabad, PAK; 3 Ophthalmology, Al-Shifa Trust Eye Hospital, Islamabad, PAK

**Keywords:** tractional retinal detachment, retinal detachment, diabetes, pars plana vitrectomy, 27 gauge

## Abstract

Objective

The objective of this article was to report the clinical and surgical outcomes of diabetic tractional retinal detachment (TRD) with 27-gauge plus pars plana vitrectomy (27G+ PPV)

Methods

This is a retrospective, consecutive cohort study of 196 eyes of 176 patients that underwent 27G+ PPV for TRD from July 2015 to June 2019 at the ophthalmology department of Shifa International Hospital, Islamabad. The outcomes include primary and secondary anatomical attachment of the retina, best-corrected visual acuity, and post-operative complications.

Results

The mean age of the patients in this study was 55.3 ± 11.3 years. Out of 176 patients, there were 47.2% (n=83) females. The mean operating time calculated was 60 ± 36 min (range 22-130 min). Of 196 eyes, 64.3% (n=126) also combined phacoemulsification with lens implantation. Internal limiting membrane peeling was done in 11.7% (n=23) of the cases. Post-operatively, 98% (n=192) achieved primary retinal attachment, and 1.5% (n=3) underwent a second procedure to achieve retinal attachment. At three months follow-up, the mean best corrected visual acuity (BCVA) remarkably improved from 1.86 ± 0.59 to 0.54 ± 0.32 logarithm of the minimal angle of resolution (logMAR) (p-value<0.001). Among complications, one patient had intra-operative suprachoroidal oil migration, which was managed successfully, while post-operatively, 11 patients (5.6%) developed a transient rise in intraocular pressure, which was controlled with anti-glaucoma drugs, and one patient had vitreous cavity hemorrhage which resolved on its own with time.

Conclusion

This study strongly suggests that the 27G+ PPV offers successful repair of eyes with diabetic TRD with statistically significant improvement in visual acuity and minimal rate of complications.

## Introduction

Tractional retinal detachment (TRD) is among the serious vision-impairing complications of diabetic retinopathy, often requiring vitrectomy. [1] Micro-incision vitrectomy surgery (MIVS) comprising of 23 and 25 gauge (G) instruments has significantly upgraded the vitrectomy procedures with less operative time, effective wound healing, reduced postoperative inflammation, and speedy visual recovery, as opposed to the conventional 20G pars plana vitrectomy (PPV) [2]. 

In 2010, Oshima et al., for the first time, reported the successful use of a further smaller, sutureless 27G vitrectomy system [3]. Since then, the 27G+ PPV has gained popularity, and many surgeons have reviewed its effectiveness, safety, and outcomes for various vitreoretinal pathologies [4-7]. Smaller data, along with analysis of 27G instrumentation on simple retinal diseases, have limited their research work. Another study was also done to explore its surgical outcomes for various complex posterior segment pathologies. However, this included the results from different surgeons [8]. 

A study reviewed the results in 665 eyes that underwent 27G+ PPV [9] and reported its safety in a versatile spectrum of posterior segment pathologies. Our study aims to evaluate the surgical and functional outcomes of 27G+ PPV exclusively in diabetic TRD. Although it is one of the most advanced diabetic eye diseases in our country, owing to its low socioeconomic status with lack of awareness and limited access to tertiary care hospitals, we come across it regularly in our clinics.

## Materials and methods

Study design and patients

This is a retrospective, consecutive cohort study performed at the Shifa International Hospital, Islamabad, where a total of 196 eyes of 176 patients were studied who had 27G+ PPV for diabetic TRD from 1st of July 2015 to 30th of June 2019 after getting Institutional Review Board (IRB) approval (Reference#219-709-2019). The medical record numbers of the patients who underwent 27G+ PPV for diabetic TRD were collected from the surgical data sheet of the operation theatre and then analyzed. All patients who had diabetic TRD involving the macula were included. Patients with prior history of retinal surgery, either PPV or scleral buckling, were excluded from this study.

Surgery was done by a single vitreoretinal surgeon after getting written informed consent from the patients. Information gathered from available medical records of eyes with diabetic TRD included age, gender, laterality of the eye, best corrected visual acuity (BCVA) using the Snellen visual acuity chart before the surgery and on 7th post-op day, one month, and then after three months post-op. Operative data included type of anesthesia, date, duration of surgery, surgical steps, tamponade agent, and intra-operative complications such as iatrogenic retinal breaks or cataracts. Re-operations were performed in the eyes that had recurrent retinal detachment (RD) or recurrent vitreous hemorrhage.

Surgical techniques

Patients were given modified retro bulbar block (1% lignocaine and 0.5% bupivacaine) and, in some cases, general anesthesia as per their requirement. Bupivacaine 0.5% was given as a sub-tenon injection in addition to the local anesthesia when needed. All the diabetic TRD patients were operated on using a 27G+ PPV Constellation Vitrectomy System (Alcon Laboratories, Fort Worth, Texas). A noncontact wide-angle viewing system (BIOM, Oculus Inc., Wetzlar, Germany) was utilized for posterior segment visualization. In every case, three pars plana sclerotomy ports with trocars inserted at 15 degrees to the sclera were made in the usual superonasal, superotemporal, and inferotemporal quadrants. These valve cannulas were placed 3.5 to 4 mm from the limbus. The cannula for infusion was attached to the inferotemporal cannula. Patients with significant cataracts underwent routine phacoemulsification with intraocular lens implantation via 2.2 mm clear corneal incisions. All cataract surgeries were performed before inserting the trocars. Every 27G+ PPV instrument, including a high-speed vitreous cutter, inner limiting membrane (ILM) forceps, diathermy probe, and endolaser probe were used. During the operation, a core vitrectomy was performed first. Removal of the tractional proliferative membranes was carried out using a vitrectomy probe by making spaces under the membranes, and 27G+ micro forceps were employed when needed. Panretinal photocoagulation was accomplished with the curved laser probe. Membrane staining dye such as MembraneBlue Dual (Dutch Ophthalmic Research Center (DORC), Zuidland, Netherlands) or ILM-BLUE dye (DORC) was used to mark and stain the epiretinal membrane or ILM before peeling it after two minutes with the ILM forceps. Vitreous substitutes as a tamponade agent used in these patients were air, intraocular gases like sulfur hexafluoride (SF6), hexafluoroethane (C2F6), octa fluoro propane (C3F8), and silicon oil (SO) of 1300 centistokes. The choice was made according to the degree of traction present and the complexity of the detached retina. Sclerotomy sites were closed with either 6-0 Vicryl sutures only in patients with continuous wound leakage after gentle massage and in cases where SO was used as an ocular tamponade. Subconjunctival antibiotics (gentamycin) and corticosteroids (dexamethasone) were given in every case at the end of the procedure. Patients were given topical 1% prednisolone acetate four to six times and 0.5% Moxifloxacin four times a day, and 1% cyclopentolate hydrochloride two times a day, postoperatively.

Statistical analysis

All data were completely arranged, tabulated, and analyzed by SPSS version 21 (IBM Inc., Armonk, New York). Percentages and frequencies were measured for qualitative figures, while the mean and standard deviation were used to assess quantitative variables. BCVA was noted using the standard Snellen visual acuity chart and then converted to the logarithm of the minimal angle of resolution (logMAR) visual acuity. Counting fingers and hand movement vision were converted to 1.98 and 2.28, respectively, as elaborated in a previous study [10]. Paired sample T-test was performed to compare preoperative and postoperative logMAR visual acuity. A p-value of <0.05 was taken as significant.

## Results

One hundred ninety-six eyes of 176 patients were included in our study, with 52.8% (n=93) being male. Their mean age was 55.3± 11.3 years. One hundred thirteen eyes had associated vitreous hemorrhage along TRD. The modified retro bulbar block was used in 96.4% (n=189) cases, while 3.5% (n=7) patients were given general anesthesia either by their own choice or because they were too young. There were no anesthesia-related complications.

**Figure 1:**
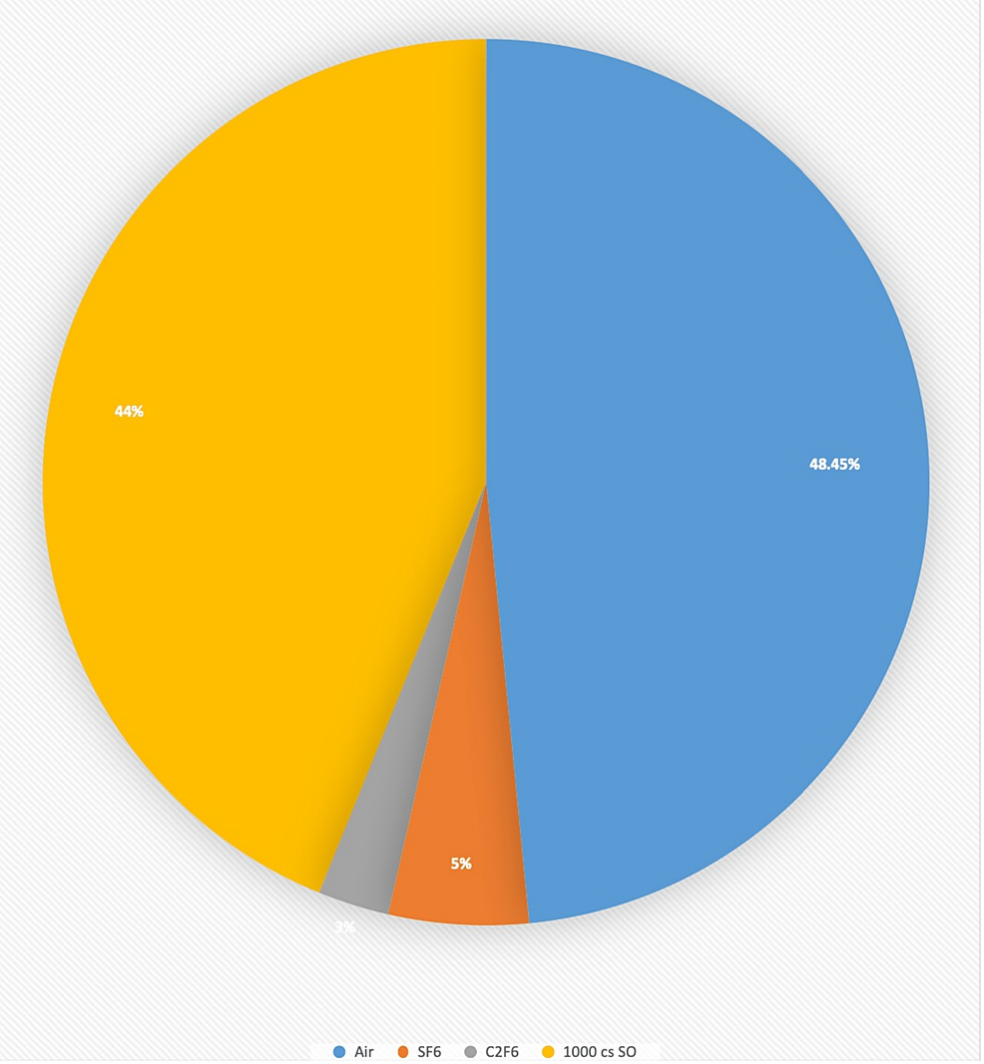
Types of tamponade agents used in diabetic TRD TRD - tractional retinal detachment, SF6 - sulfur hexafluoride, C2F6 - hexa fluoroethane, SO - silicone oil

27G+ PPV was carried out in all the cases, while 64.3% (n=126) of eyes also underwent a combined phacoemulsification and lens implantation. The mean operating time was 60 ± 26 min (range 22-130 min). Some additional important steps were carried out in required cases as well. ILM peeling was done in 11.7% (n=23) of surgeries. At the end of the procedure, tamponade agents used were air in 48.5% (n= 95), SO in 44% (n=86), SF6 in 5% (n=10), and C2F6 gas in 3% (n=5) of the patients (Figure 1).

Conversion to larger 23 or 25G instruments was not required in any of the cases except for SO injection at the end of the surgery, where a 25G trocar was employed. The wounds did not require suturing unless a persistent leakage was noted, and in cases where silicone oil was used as a tamponade, 44% (n=86). 

BCVA of the patients was noted at one week, one month, and finally three months post-op. Visual outcomes were compared with those before the surgery with three months post-op, and surgical outcomes were noted. Ninety-eight percent (n=192) achieved retinal reattachment with a single surgery, and 1.5% (n=3) had second surgery done with SO as a tamponade to attain final retinal reattachment (Table 1). 

**Table 1:** Anatomical outcome of 27G+ PPV in diabetic TRD 27G+ PPV - 27 gauge plus pars plana vitrectomy, TRD - tractional retinal detachment, SO - silicone oil, VCH - vitreous cavity hemorrhage

Diabetic TRD (196 eyes)	Primary result	Further management	Final result
Attached	192 (98%)		
Re-detached	3 (1.5%)	27G+ PPV, SO (n=3)	Attached
VCH	1 (0.5%)	Observation	Resolved

Mean BCVA remarkably improved from 1.86± 0.59 logMAR before surgery to 0.54 ± 0.32 three months after surgery (p<0.001) (Table 2).

**Table 2:** Comparison of preoperative and postoperative BCVA in 27G+ PPV TRD BCVA - best corrected visual acuity, 27G+ PPV - 27 gauge plus pars plana vitrectomy, TRD - tractional retinal detachment

Surgical indication (no of eyes)	Preoperative BCVA (mean logMAR ± SD)	Postoperative BCVA (mean logMAR ± SD)	p-value
Diabetic TRD (196)	1.86 ± 0.59	0.54 ± 0.32	<0.001

Out of 196 eyes operated on, 3% (n=6) developed iatrogenic retinal breaks that were managed effectively. Intraoperatively, one patient developed suprachoroidal oil migration, which was successfully managed surgically. Postoperative complications included a transient rise in intraocular pressure in eleven patients, which was timely controlled with anti-glaucoma drugs, and another patient had vitreous cavity hemorrhage, which resolved on its own with time. SO-filled eyes underwent another procedure to remove the oil after three to four months when complete attachment of the retina was ensured.

## Discussion

One of the vision-threatening complications of proliferative diabetic retinopathy in patients with poor glycemic control is TRD, for which PPV is the most appropriate surgical approach if the macula is involved. Recently, 27G PPV has gained attention in the treatment of different retinal pathologies. With less operating time, a high-speed vitreous cutter allowing easy removal of membranes, sutureless surgery without postoperative hypotonic eyes, fewer postoperative complications, and stable BCVA, this advancement in MIVS is groundbreaking.

Thus, the conventional 20G vitrectomy system is no longer a surgeon's choice because 23, 25, and now with further improvement, 27G vitrectomy instruments have been proven more efficient and safe [1-4]. The literature has previously reported that smaller gauges have a higher cut rate, better fluidics, and fewer chances of iatrogenic breaks [11,12].

Since the initiation of the 27G vitrectomy system in 2010 by Oshima et al., different surgeons have done studies to report the outcomes of this system in various posterior segment diseases as their confidence in this system grew [3,5,11,12]. However, less work has been done regarding its outcomes in complex pathologies like diabetic TRD (Table 3). Our paper, in comparison, is the only study that is done on a large number of TRD patients who had 27G+ PPV in adequate operating time, with minimum complication rate and good BCVA at three months of follow-up. 

**Table 3:** Comparison of different studies with 27G PPV in diabetic TRD 27G PPV - 27 gauge pars plana vitrectomy, TRD - tractional retinal detachment, BCVA - best corrected visual acuity

The study, publication year	The sample size used (eyes with TRD)	Operating time (min)	Postoperative BCVA (mean logMAR ± SD)	Postoperative complications, n (%)
Chen et al, 2021 [12]	21	56.7 ± 19.6	0.79 ± 0.55	8 (38.1)
Cruziñigo et al, 2017 [13]	12	50± 5	0.92 ± 0.40	3 (25)
Oshima et.al, 2021 [12]	42	77 ± 37	0.46 ± 0.51	10 (23.8)
Present study	196	60 ± 26	0.54 ± 0.32	2 (1)

We found the 27G+ vitrectomy instruments efficient and rigid enough to pass through tight spaces of tractional membranes, offering quick and simplified removal of membranes as opposed to Yoneda, who considered them fragile. [11]

Chen et al., in their study, showed that due to the smaller size of the vitrector and better fluidics, it is possible to use the suction of the cutter to peel away the membranes in certain scenarios without using the membrane peeling forceps. He noted that there was less exchange of instruments during the surgery, effective removal of vitreous gel close to a detached retina, and operative time was similar to that with a 25G vitrectomy system. [12] We noted similar ease and better surgical experience with a smaller 27G system as well.

Previously done studies and literature have shown SF6 and C3F8 as effective tamponades during 27G+ for diabetic TRD patients. SO was employed in a few cases only. These studies had the limitation of having a small sample size. [12-15] However, we studied a large sample of TRD patients and demonstrated that air, SO, and other tamponade agents can also be effectively used in diabetic TRD patients depending on the extent and severity of pathology.

The comparative study by Chen et al. was conducted on 21 eyes, and a 38 % post-procedural complication rate was noted. [12] Oshima et al. also, in their pilot survey, depicted that 42 patients showed improvement in BCVA who had 27G+ vitrectomy but had a 23.8% postoperative complication percentage. [14] Similarly, 12 eyes were operated on using 27 gauge vitrectomy and studied retrospectively by Cruz-Iñigo et al. showed similar results. [13]

However, our study, in comparison, studied 196 cases where BCVA was observed to be significantly improved, with one patient developing intraoperatively suprachoroidal hemorrhage and only two cases presenting with postoperative complications. One had raised intraocular pressure, which was safely controlled with pressure-lowering drugs, and the other had vitreous cavity hemorrhage, an eminent postoperative complication. [12] Three eyes required reoperation with silicon oil as tamponade, and final reattachment was acquired.

This research paper, with all its strengths, has the limitations of being a retrospective, noncomparative analysis of patients from a single hospital setup, with patients who were treated surgically for TRD with 27G+ PPV. Also, a long-term follow-up of patients would have given surgeons a better insight regarding the rate of complications and disease prognosis.

## Conclusions

According to our literature review, our work ranks among one of the largest studies done worldwide to evaluate the results of 27G+ PPV on a specific retinal pathology, i.e., diabetic TRD involving the macula. With its large sample size, minimal post-procedure complications, good anatomical attachment rate as well as favorable post-op mean BCVA, and satisfactory wound closure, our paper has positively shown the 27G+ PPV to be safe and effective for the repair of complicated procedures like diabetic TRD.
